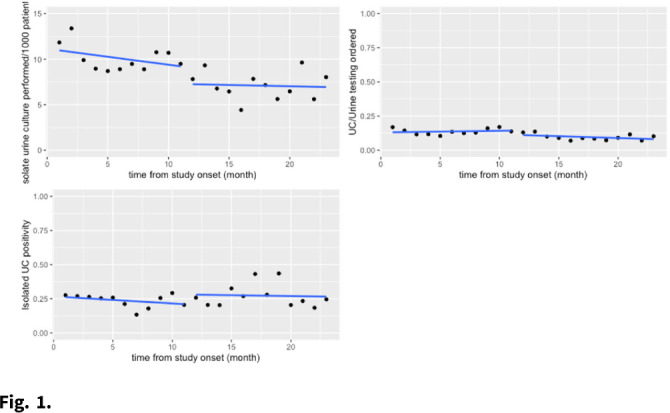# Changing the use of isolated urine-culture testing with diagnostic testing stewardship

**DOI:** 10.1017/ash.2022.163

**Published:** 2022-05-16

**Authors:** Jessica Penney, Angie Rodday, Paola Sebastiani, David Snydman, Shira Doron

## Abstract

**Background:** Urine testing is one of the more frequently ordered diagnostic tests among hospitalized patients. Many hospitals have implemented urinalysis with reflex culture (UARC) as a method of diagnostic testing stewardship to guide appropriate use of urine testing. Isolated urine culture, or urine culture without preceding urinalysis, is the most appropriate diagnostic test for patients who are neutropenic, pregnant, or those about to undergo an invasive urologic procedures. This testing is often used beyond these indications in hospitals though, potentially leading to overdiagnosis of UTI and overtreatment of asymptomatic bacteriuria. **Methods:** We compared outcomes in the preimplementation period (December 2018–November 2019) to those in the postintervention period (December 2019–October 2020) at an academic medical center. The intervention was the addition of an indication selection (ie pregnancy, neutropenia, etc) to the isolated urine-culture order in the electronic medical record (EMR). The primary outcomes were isolated urine culture rate per 1,000 patient days and urine-culture positivity. Our exploratory analysis included a review of selected indications after the intervention was implemented and a chart review of a subset of these tests for appropriateness. The primary analysis was performed using interrupted time-series negative binomial regression. **Results:** There was no significant change in isolated urine-culture rates after the intervention (11.18 cultures per 1,000 patient days before the intervention versus to 7.75 cultures per 1,000 patient days after the intervention; *P* > .90), and there were as no significant pre- or postintervention trends. We detected no significant change in isolated urine-culture positivity: 26.9% before the intervention versus 26.7% after the intervention (*P* > .90). These results are shown graphically in Fig. 1. In the exploratory analysis, of 661 isolated urine-culture tests ordered in the postintervention period, the indication for testing was left blank in 71.9% of tests. The other most common reasons for testing included other (16%), pregnancy (5.7%), and neutropenia (4.4%). In the 100 tests reviewed for appropriateness, only 8% had a documented diagnosis corresponding with the selected indication for testing. **Discussion:** The addition of an indication selection for isolated urine culture testing did not change the rates of culture ordering or the culture’s subsequent likelihood of positivity. In the exploratory analysis, most providers were incorrectly selecting this testing rather than UARC as prompted. Next steps could potentially be removing the “other” category and requiring a selected answer or requiring approval from stewardship team prior to ordering. Continued education of providers is paramount to the appropriate use of diagnostic testing.

**Funding:** None

**Disclosures:** None

**Figure:**